# Supplementary material for: Seasonal Variations of Rosmarinic Acid and Its Glucoside and Expression of Genes Related to Their Biosynthesis in Two Medicinal and Aromatic Species of Salvia subg. Perovskia
Source: Biology (Basel). 2021 May 22;10(6):458. doi: 10.3390/biology10060458 (PMC8224735; doi:10.3390/biology10060458)
Supplement: Supplementary file 1 [file biology-10-00458-s001.zip › biology-1203623.pdf]

Supplementary material

Seasonal variations of rosmarinic acid and its glucoside and expression of genes related to their biosynthesis in two medicinal and aromatic species of Salvia subg. Perovskia

Marta Stafiniak, Sylwester Ślusarczyk, Bartosz Pencakowski, Adam Matkowski, Mehdi Rahimmalek, Monika Bielecka

Table S1. Selected climate features for the period 1986-2005 and for 2014, 2015 and 2016 (after *Index Seminum* 2016, ISSN 1733–9006).

Climatic features	Measures 1986-2005	2014	2015	2016
Mean annual air temperature [°C]	8.9	11.7	11.1	(11.3)*
Mean air temperature - January [°C]	- 0.4	1.0	2.9	- 0.3
Mean air temperature - July [°C]	18.9	21.9	20.9	20.3
Absolute maximum air temperature [°C]	37.4	33.8	37.9	35.1
Absolute minimum air temperature [°C]	- 32.0	- 13.0	- 7.4	(- 15.8)*
Annual sunlight [hours]	1755.2	1917.1	2159.4	(1836.7)*
Annual vegetation period [days]	191.5	213	187	228
Annual number of frostfree [days]	129.1	140	150	159
Annual sum of precipitation [mm]	519	572.8	388.3	(586.0)*
Number of the precipitation days [days]	157.5	148	140	(155)*

*without XII 2016

Table S2. The content of salvianolic acid L [mg/g d.w.±SD] as rosmarinic acid equivalents in roots of *S. abrotanoides* and *S. yangii* at the start of the vegetation season (SOS), middle of the season (MOS), and end of the season (EOS). Statistical significance of differences in chemical parameters between samples was evaluated with one-way ANOVA with post-hoc Tukey's multiple comparison tests.

	Salvia abrotanoides			***Salvia yangii***		
**Compound**	**SOS**	**MOS**	**EOS**	**SOS**	**MOS**	**EOS**
			roots			
Salvianolic acid L	0.50 ± 0.07	0.32 ± 0.06^{a}	0.00 ± 0.00^{a}	0.62 ± 0.02	0.99 ± 0.07^{A,B}	0.43±0.06^{a,B}

a - significant differences in comparison to SOS, b - significant differences in comparison to *S. abrotanoides*, c - significant differences in comparison to leaves, $p < 0.05$ lower case font, $p < 0.001$ italic font, $p < 0.0001$ upper case font, SOS – start of season, MOS – middle of season, EOS – end of season

Table S3. Normalized expression (NE) of rosmarinic acid biosynthesis pathway genes in leaves and roots of *S. abrotanoides* and *S. yangii* in three growth stages during the vegetative season.

cDNA No.	Sample name	Gene	Stage of growth	Plant organ	Species	NE	Std.Dev. NE
1	SOS SA L	*TAT*	SOS	L	SA	8.60	0.74
3	SOS SY L	*TAT*			SY	6.50	0.47
5	MOS SA L	*TAT*	MOS	L	SA	1.16	0.26
8	MOS SY L	*TAT*			SY	0.55	0.04
11	EOS SA L	*TAT*	EOS	L	SA	0.95	0.27
13	EOS SY L	*TAT*			SY	0.35	0.03
2	SOS SA R	*TAT*	SOS	R	SA	6.55	1.20
4	SOS SY R	*TAT*			SY	1.64	0.41
6	MOS SA R	*TAT*	MOS	R	SA	7.00	1.86
9	MOS SY R	*TAT*			SY	1.24	0.16
12	EOS SA R	*TAT*	EOS	R	SA	0.23	0.03
14	EOS SY R	*TAT*			SY	0.16	0.08
1	SOS SA L	*HPPR*	SOS	L	SA	4.95	0.52
3	SOS SY L	*HPPR*			SY	2.85	0.20
5	MOS SA L	*HPPR*	MOS	L	SA	2.22	0.57
8	MOS SY L	*HPPR*			SY	1.60	0.10
11	EOS SA L	*HPPR*	EOS	L	SA	2.10	0.60
13	EOS SY L	*HPPR*			SY	1.82	0.15
2	SOS SA R	*HPPR*	SOS	R	SA	0.50	0.12
4	SOS SY R	*HPPR*			SY	0.17	0.04
6	MOS SA R	*HPPR*	MOS	R	SA	0.52	0.15
9	MOS SY R	*HPPR*			SY	0.20	0.03
12	EOS SA R	*HPPR*	EOS	R	SA	0.16	0.02
14	EOS SY R	*HPPR*			SY	0.12	0.06
1	SOS SA L	*PAL*	SOS	L	SA	8.50	0.80
3	SOS SY L	*PAL*			SY	4.91	0.48
5	MOS SA L	*PAL*	MOS	L	SA	0.48	0.11
8	MOS SY L	*PAL*			SY	0.37	0.04
11	EOS SA L	*PAL*	EOS	L	SA	1.39	0.41
13	EOS SY L	*PAL*			SY	0.52	0.05
2	SOS SA R	*PAL*	SOS	R	SA	4.08	0.75
4	SOS SY R	*PAL*			SY	0.58	0.17
6	MOS SA R	*PAL*	MOS	R	SA	2.96	0.87
9	MOS SY R	*PAL*			SY	0.90	0.15
12	EOS SA R	*PAL*	EOS	R	SA	0.38	0.08
14	EOS SY R	*PAL*			SY	0.33	0.17
1	SOS SA L	*C4H*	SOS	L	SA	12.37	1.75
3	SOS SY L	*C4H*			SY	8.23	1.10
5	MOS SA L	*C4H*	MOS	L	SA	1.65	0.37

8	MOS SY L	*C4H*			SY	1.30	0.13
11	EOS SA L	*C4H*	EOS	L	SA	2.44	0.70
13	EOS SY L	*C4H*			SY	1.31	0.16
2	SOS SA R	*C4H*	SOS	R	SA	6.41	1.14
4	SOS SY R	*C4H*			SY	1.90	0.46
6	MOS SA R	*C4H*	MOS	R	SA	5.88	1.55
9	MOS SY R	*C4H*			SY	1.73	0.25
12	EOS SA R	*C4H*	EOS	R	SA	0.52	0.07
14	EOS SY R	*C4H*			SY	0.61	0.32
1	SOS SA L	*4CL*	SOS	L	SA	0.43	0.13
3	SOS SY L	*4CL*			SY	0.28	0.03
5	MOS SA L	*4CL*	MOS	L	SA	0.15	0.04
8	MOS SY L	*4CL*			SY	0.08	0.01
11	EOS SA L	*4CL*	EOS	L	SA	0.48	0.14
13	EOS SY L	*4CL*			SY	0.16	0.02
2	SOS SA R	*4CL*	SOS	R	SA	1.07	0.26
4	SOS SY R	*4CL*			SY	0.33	0.09
6	MOS SA R	*4CL*	MOS	R	SA	1.09	0.46
9	MOS SY R	*4CL*			SY	0.15	0.08
12	EOS SA R	*4CL*	EOS	R	SA	0.19	0.04
14	EOS SY R	*4CL*			SY	0.10	0.05
1	SOS SA L	*RAS1*	SOS	L	SA	3.09	0.35
3	SOS SY L	*RAS1*			SY	2.20	0.14
5	MOS SA L	*RAS1*	MOS	L	SA	1.62	0.37
8	MOS SY L	*RAS1*			SY	1.57	0.20
11	EOS SA L	*RAS1*	EOS	L	SA	1.03	0.29
13	EOS SY L	*RAS1*			SY	0.93	0.08
2	SOS SA R	*RAS1*	SOS	R	SA	1.50	0.27
4	SOS SY R	*RAS1*			SY	0.21	0.05
6	MOS SA R	*RAS1*	MOS	R	SA	0.97	0.26
9	MOS SY R	*RAS1*			SY	0.32	0.04
12	EOS SA R	*RAS1*	EOS	R	SA	0.34	0.05
14	EOS SY R	*RAS1*			SY	0.11	0.06
1	SOS SA L	*RAS2*	SOS	L	SA	0.2	0.03
3	SOS SY L	*RAS2*			SY	0.22	0.04
5	MOS SA L	*RAS2*	MOS	L	SA	0.32	0.07
8	MOS SY L	*RAS2*			SY	0.24	0.02
11	EOS SA L	*RAS2*	EOS	L	SA	0.02	0.00474
13	EOS SY L	*RAS2*			SY	0.01	0.00183
2	SOS SA R	*RAS2*	SOS	R	SA	0.01	0.00202
4	SOS SY R	*RAS2*			SY	0.00073	0.000508
6	MOS SA R	*RAS2*	MOS	R	SA	0.00294	0.00097
9	MOS SY R	*RAS2*			SY	0.000272	0.0000681
12	EOS SA R	*RAS2*	EOS	R	SA	0.000206	0.000119
14	EOS SY R	*RAS2*			SY	0.00216	0.00125

1	SOS SA L	*CYP98A14*	SOS	L	SA	4.5	1.11
3	SOS SY L	*CYP98A14*			SY	3.52	1.6
5	MOS SA L	*CYP98A14*	MOS	L	SA	0.77	0.17
8	MOS SY L	*CYP98A14*			SY	0.6	0.17
11	EOS SA L	*CYP98A14*	EOS	L	SA	0.4	0.12
13	EOS SY L	*CYP98A14*			SY	0.71	0.17
2	SOS SA R	*CYP98A14*	SOS	R	SA	1.55	0.42
4	SOS SY R	*CYP98A14*			SY	0.18	0.06
6	MOS SA R	*CYP98A14*	MOS	R	SA	1.11	0.35
9	MOS SY R	*CYP98A14*			SY	0.13	0.05
12	EOS SA R	*CYP98A14*	EOS	R	SA	0.12	0.04
14	EOS SY R	*CYP98A14*			SY	0.01	0.02

SOS, start of season; MOS, middle of season; EOS, end of season; SA, *S. abrotanoides*; SY, *S. yangii*; L, leaves; R, roots; NE, normalized expression

Table S4. Statistical significance of differences in chemical parameters between samples and significance of differences between means of expression data sets determined with one-way ANOVA with post-hoc Tukey's multiple comparison tests.

Samples compared	*TAT*		*HPPR*		*PAL*		*C4H*		*4CL*		*RAS1*		*RAS2*		*CYP*		RA (Rosmarinic Acid)		SF (Salviaflaside)	
	Summary	Adjusted P Value	Summary	Adjusted P Value	Summary	Adjusted P Value	Summary	Adjusted P Value	Summary	Adjusted P Value	Summary	Adjusted P Value	Summary	Adjusted P Value	Summary	Adjusted P Value	Summary	Adjusted P Value	Summary	Adjusted P Value
SOS SA L vs. SOS SY L	*	0.0471	****	<0.0001	****	<0.0001	***	0.0003	ns	0.992	**	0.0018	ns	0.9973	ns	0.6717	****	<0.0001		
SOS SA L vs. MOS SA L	****	<0.0001	****	<0.0001	****	<0.0001	****	<0.0001	ns	0.6614	****	<0.0001	***	0.0003	****	<0.0001	ns	0.5436		
SOS SA L vs. MOS SY L	****	<0.0001	****	<0.0001	****	<0.0001	****	<0.0001	ns	0.3544	****	<0.0001	ns	0.7384	****	<0.0001	ns	0.9988		
SOS SA L vs. EOS SA L	****	<0.0001	****	<0.0001	****	<0.0001	****	<0.0001	ns	>0.9999	****	<0.0001	****	<0.0001	****	<0.0001	**	0.0028		
SOS SA L vs. EOS SY L	****	<0.0001	****	<0.0001	****	<0.0001	****	<0.0001	ns	0.706	****	<0.0001	****	<0.0001	****	<0.0001	*	0.0226		
SOS SA L vs. SOS SA R	ns	0.0567	****	<0.0001	****	<0.0001	****	<0.0001	**	0.0043	****	<0.0001	****	<0.0001	***	0.0001	****	<0.0001		
SOS SA L vs. SOS SY R	****	<0.0001	****	<0.0001	****	<0.0001	****	<0.0001	ns	0.9998	****	<0.0001	****	<0.0001	****	<0.0001	ns	0.4143		
SOS SA L vs. MOS SA R	ns	0.2537	****	<0.0001	****	<0.0001	****	<0.0001	**	0.003	****	<0.0001	****	<0.0001	****	<0.0001	***	0.0009		
SOS SA L vs. MOS SY R	****	<0.0001	****	<0.0001	****	<0.0001	****	<0.0001	ns	0.6614	****	<0.0001	****	<0.0001	****	<0.0001	ns	0.8037		
SOS SA L vs. EOS SA R	****	<0.0001	****	<0.0001	****	<0.0001	****	<0.0001	ns	0.8265	****	<0.0001	****	<0.0001	****	<0.0001	ns	0.75		
SOS SA L vs. EOS SY R	****	<0.0001	****	<0.0001	****	<0.0001	****	<0.0001	ns	0.4356	****	<0.0001	****	<0.0001	****	<0.0001	ns	0.1039		
SOS SY L vs. MOS SA L	****	<0.0001	ns	0.341	****	<0.0001	****	<0.0001	ns	0.9975	ns	0.097	**	0.0032	***	0.0004	****	<0.0001		
SOS SY L vs. MOS SY L	****	<0.0001	**	0.0015	****	<0.0001	****	<0.0001	ns	0.9375	ns	0.0538	ns	0.9973	***	0.0002	****	<0.0001		
SOS SY L vs. EOS SA L	****	<0.0001	ns	0.1471	****	<0.0001	****	<0.0001	ns	0.9375	****	<0.0001	****	<0.0001	****	<0.0001	****	<0.0001		
SOS SY L vs. EOS SY L	****	<0.0001	*	0.0125	****	<0.0001	****	<0.0001	ns	0.9988	****	<0.0001	****	<0.0001	***	0.0003	****	<0.0001		
SOS SY L vs. SOS SA R	ns	>0.9999	****	<0.0001	ns	0.5364	ns	0.3473	***	0.0003	*	0.0224	****	<0.0001	*	0.0174	**	0.0025		
SOS SY L vs. SOS SY R	****	<0.0001	****	<0.0001	****	<0.0001	****	<0.0001	ns	>0.9999	****	<0.0001	****	<0.0001	****	<0.0001	****	<0.0001		
SOS SY L vs. MOS SA R	ns	0.9989	****	<0.0001	**	0.001	ns	0.0911	***	0.0002	****	<0.0001	****	<0.0001	**	0.002	***	0.0002		
SOS SY L vs. MOS SY R	****	<0.0001	****	<0.0001	****	<0.0001	****	<0.0001	ns	0.9975	****	<0.0001	****	<0.0001	****	<0.0001	****	<0.0001		
SOS SY L vs. EOS SA R	****	<0.0001	****	<0.0001	****	<0.0001	****	<0.0001	ns	>0.9999	****	<0.0001	****	<0.0001	****	<0.0001	****	<0.0001		
SOS SY L vs. EOS SY R	****	<0.0001	****	<0.0001	****	<0.0001	****	<0.0001	ns	0.9688	****	<0.0001	****	<0.0001	****	<0.0001	****	<0.0001		
MOS SA L vs. MOS SY L	ns	0.9941	ns	0.3624	ns	>0.9999	ns	>0.9999	ns	>0.9999	ns	>0.9999	*	0.0302	ns	>0.9999	ns	0.137		
MOS SA L vs. EOS SA L	ns	>0.9999	ns	>0.9999	ns	0.4066	ns	0.9907	ns	0.4356	ns	0.0865	****	<0.0001	ns	0.9996	****	<0.0001		
MOS SA L vs. EOS SY L	ns	0.9521	ns	0.8774	ns	>0.9999	ns	>0.9999	ns	>0.9999	*	0.0255	****	<0.0001	ns	>0.9999	***	0.0001		
MOS SA L vs. SOS SA R	****	<0.0001	****	<0.0001	****	<0.0001	****	<0.0001	****	<0.0001	ns	0.9999	****	<0.0001	ns	0.8873	****	<0.0001		
MOS SA L vs. SOS SY R	ns	0.9993	****	<0.0001	ns	>0.9999	ns	>0.9999	ns	0.9688	****	<0.0001	****	<0.0001	ns	0.9816	ns	>0.9999		
MOS SA L vs. MOS SA R	****	<0.0001	****	<0.0001	****	<0.0001	***	0.0002	****	<0.0001	*	0.0421	****	<0.0001	ns	0.9998	****	<0.0001		
MOS SA L vs. MOS SY R	ns	>0.9999	****	<0.0001	ns	0.9894	ns	>0.9999	ns	>0.9999	****	<0.0001	****	<0.0001	ns	0.9671	ns	>0.9999		
MOS SA L vs. EOS SA R	ns	0.8894	****	<0.0001	ns	>0.9999	ns	0.8944	ns	>0.9999	****	<0.0001	****	<0.0001	ns	0.9634	*	0.0154		
MOS SA L vs. EOS SY R	ns	0.8375	****	<0.0001	ns	>0.9999	ns	0.9354	ns	>0.9999	****	<0.0001	****	<0.0001	ns	0.9026	ns	0.9955		
MOS SY L vs. EOS SA L	ns	0.9999	ns	0.6589	ns	0.2579	ns	0.8891	ns	0.1946	ns	0.1509	****	<0.0001	ns	>0.9999	*	0.0223		
MOS SY L vs. EOS SY L	ns	>0.9999	ns	0.9984	ns	>0.9999	ns	>0.9999	ns	>0.9999	*	0.0477	****	<0.0001	ns	>0.9999	ns	0.1439		
MOS SY L vs. SOS SA R	****	<0.0001	**	0.0064	****	<0.0001	****	<0.0001	****	<0.0001	ns	>0.9999	****	<0.0001	ns	0.7094	***	0.0005		
MOS SY L vs. SOS SY R	ns	0.7564	***	0.0002	ns	>0.9999	ns	0.9991	ns	0.7892	****	<0.0001	****	<0.0001	ns	0.9989	ns	0.0897		
MOS SY L vs. MOS SA R	****	<0.0001	**	0.0077	****	<0.0001	****	<0.0001	****	<0.0001	ns	0.077	****	<0.0001	ns	0.994	**	0.0076		

MOS SY L vs. MOS SY R	ns	0.9844	***	0.0003	ns	0.9446	ns	>0.9999	ns	>0.9999	****	<0.0001	****	<0.0001	ns	0.997	ns	0.2944		
MOS SY L vs. EOS SA R	ns	>0.9999	***	0.0002	ns	>0.9999	ns	0.9916	ns	0.9994	****	<0.0001	****	<0.0001	ns	0.9964	ns	0.9956		
MOS SY L vs. EOS SY R	ns	0.9999	***	0.0002	ns	>0.9999	ns	0.9969	ns	>0.9999	****	<0.0001	****	<0.0001	ns	0.9816	*	0.0154		
EOS SA L vs. EOS SY L	ns	0.9948	ns	0.9885	ns	0.4699	ns	0.8944	ns	0.4792	ns	>0.9999	ns	>0.9999	ns	>0.9999	ns	0.9987		
EOS SA L vs. SOS SA R	****	<0.0001	****	<0.0001	****	<0.0001	***	0.0005	*	0.0102	ns	0.3006	ns	>0.9999	ns	0.4533	ns	0.8902		
EOS SA L vs. SOS SY R	ns	0.9844	****	<0.0001	ns	0.5703	ns	0.9997	ns	0.992	**	0.0046	ns	0.9981	ns	>0.9999	****	<0.0001		
EOS SA L vs. MOS SA R	****	<0.0001	****	<0.0001	*	0.0118	**	0.0028	**	0.0072	ns	>0.9999	ns	0.9993	ns	0.9351	ns	>0.9999		
EOS SA L vs. MOS SY R	ns	>0.9999	****	<0.0001	ns	0.9671	ns	0.9961	ns	0.4356	*	0.0197	ns	0.9976	ns	>0.9999	****	<0.0001		
EOS SA L vs. EOS SA R	ns	0.9787	****	<0.0001	ns	0.2697	ns	0.2787	ns	0.6158	*	0.0255	ns	0.9976	ns	>0.9999	ns	0.1845		
EOS SA L vs. EOS SY R	ns	0.9593	****	<0.0001	ns	0.2145	ns	0.34	ns	0.2506	**	0.0012	ns	0.999	ns	0.9994	****	<0.0001		
EOS SY L vs. SOS SA R	****	<0.0001	***	0.0007	****	<0.0001	****	<0.0001	****	<0.0001	ns	0.1087	ns	>0.9999	ns	0.8334	ns	0.3899		
EOS SY L vs. SOS SY R	ns	0.5443	****	<0.0001	ns	>0.9999	ns	0.9992	ns	0.9792	*	0.0173	ns	>0.9999	ns	0.9919	****	<0.0001		
EOS SY L vs. MOS SA R	****	<0.0001	***	0.0009	****	<0.0001	****	<0.0001	****	<0.0001	ns	>0.9999	ns	>0.9999	ns	0.9993	ns	0.9632		
EOS SY L vs. MOS SY R	ns	0.914	****	<0.0001	ns	0.9952	ns	>0.9999	ns	>0.9999	ns	0.0684	ns	>0.9999	ns	0.9838	***	0.0003		
EOS SY L vs. EOS SA R	ns	>0.9999	****	<0.0001	ns	>0.9999	ns	0.9907	ns	>0.9999	ns	0.0865	ns	>0.9999	ns	0.9816	ns	0.6476		
EOS SY L vs. EOS SY R	ns	>0.9999	****	<0.0001	ns	>0.9999	ns	0.9965	ns	>0.9999	**	0.0046	ns	>0.9999	ns	0.9406	****	<0.0001		
SOS SA R vs. SOS SY R	****	<0.0001	ns	0.9621	****	<0.0001	****	<0.0001	***	0.0007	****	<0.0001	ns	>0.9999	ns	0.2263	****	<0.0001	**	0.0032
SOS SA R vs. MOS SA R	ns	0.9996	ns	>0.9999	ns	0.1601	ns	0.9997	ns	>0.9999	ns	0.1677	ns	>0.9999	ns	0.9983	ns	0.9887	**	0.0011
SOS SA R vs. MOS SY R	****	<0.0001	ns	0.9806	****	<0.0001	****	<0.0001	****	<0.0001	****	<0.0001	ns	>0.9999	ns	0.1886	****	<0.0001	ns	0.6288
SOS SA R vs. EOS SA R	****	<0.0001	ns	0.9538	****	<0.0001	****	<0.0001	****	<0.0001	****	<0.0001	ns	>0.9999	ns	0.1817	**	0.0057	****	<0.0001
SOS SA R vs. EOS SY R	****	<0.0001	ns	0.908	****	<0.0001	****	<0.0001	****	<0.0001	****	<0.0001	ns	>0.9999	ns	0.1182	****	<0.0001	****	<0.0001
SOS SY R vs. MOS SA R	****	<0.0001	ns	0.9443	****	<0.0001	***	0.0004	***	0.0005	*	0.0102	ns	>0.9999	ns	0.7339	****	<0.0001	ns	0.9801
SOS SY R vs. MOS SY R	ns	0.9999	ns	>0.9999	ns	0.9989	ns	>0.9999	ns	0.9688	ns	>0.9999	ns	>0.9999	ns	>0.9999	ns	>0.9999	***	0.0003
SOS SY R vs. EOS SA R	ns	0.4188	ns	>0.9999	ns	>0.9999	ns	0.72	ns	0.9954	ns	0.9998	ns	>0.9999	ns	>0.9999	**	0.0094	****	<0.0001
SOS SY R vs. EOS SY R	ns	0.3522	ns	>0.9999	ns	0.9999	ns	0.7916	ns	0.8603	ns	>0.9999	ns	>0.9999	ns	>0.9999	ns	0.9994	****	<0.0001
MOS SA R vs. MOS SY R	****	<0.0001	ns	0.9693	***	0.0005	***	0.0002	****	<0.0001	*	0.0421	ns	>0.9999	ns	0.6717	****	<0.0001	***	0.0001
MOS SA R vs. EOS SA R	****	<0.0001	ns	0.9335	****	<0.0001	****	<0.0001	****	<0.0001	ns	0.0538	ns	>0.9999	ns	0.659	ns	0.0749	****	<0.0001
MOS SA R vs. EOS SY R	****	<0.0001	ns	0.8774	****	<0.0001	****	<0.0001	****	<0.0001	**	0.0027	ns	>0.9999	ns	0.5162	****	<0.0001	****	<0.0001
MOS SY R vs. EOS SA R	ns	0.8292	ns	>0.9999	ns	0.951	ns	0.8475	ns	>0.9999	ns	>0.9999	ns	>0.9999	ns	>0.9999	*	0.0411	****	<0.0001
MOS SY R vs. EOS SY R	ns	0.7661	ns	>0.9999	ns	0.9135	ns	0.8996	ns	>0.9999	ns	0.9848	ns	>0.9999	ns	>0.9999	ns	0.9391	***	0.0005
EOS SA R vs. EOS SY R	ns	>0.9999	ns	>0.9999	ns	>0.9999	ns	>0.9999	ns	>0.9999	ns	0.9706	ns	>0.9999	ns	>0.9999	**	0.0014	****	<0.0001

.
