# Supplementary figures and images for: Seasonal Variations of Rosmarinic Acid and Its Glucoside and Expression of Genes Related to Their Biosynthesis in Two Medicinal and Aromatic Species of Salvia subg. Perovskia
Source: Biology (Basel). 2021 May 22;10(6):458. doi: 10.3390/biology10060458 (PMC8224735; doi:10.3390/biology10060458)

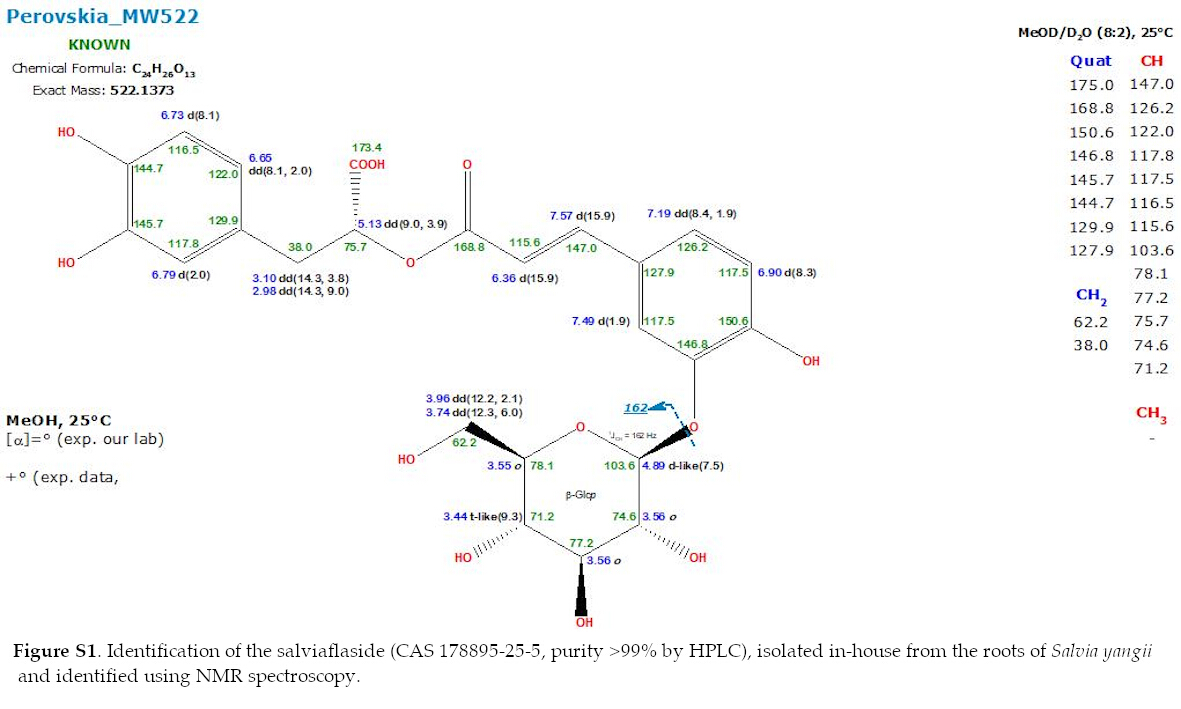

Supplement: Supplementary file 1 [file biology-10-00458-s001.zip › biology-1203623.jpg]
